# Effect of Cyclic Precalcification of Nanotubular TiO_**2**_ Layer on the Bioactivity of Titanium Implant

**DOI:** 10.1155/2013/293627

**Published:** 2013-08-29

**Authors:** Il Song Park, Eun Jin Yang, Tae Sung Bae

**Affiliations:** Department of Dental Biomaterials and Institute of Oral Bioscience, School of Dentistry, Chonbuk National University, 664-14 Deokjin-dong, Jeonju 561-756, Republic of Korea

## Abstract

The objective of this study is to investigate the effect of cyclic precalcification treatment to impart bioactive properties for titanium implants. Before precalcification, the titanium implants were subjected to blasting using hydroxyapatite (HAp), a resorbable blasting medium (RBM treated), and anodized using an electrolyte containing glycerol, H_2_O, and NH_4_F. Precalcification treatment was performed by two different methods, namely, continuous immersion treatment (CIT) and alternate immersion treatment (AIT). In CIT, the RBM treated and anodized titanium implants were immersed in 0.05 M NaH_2_PO_4_ solution at 80°C and saturated Ca(OH)_2_ solution at 100°C for 20 min, whereas during AIT, they were immersed alternatively in both solutions for 1 min for 20 cycles. Anodizing of the titanium implants enables the formation of self-organized TiO_2_ nanotubes. Cyclic precalcification treatment imparts a better bioactive property and enables an increase in activation level of the titanium implants. The removal torque values of the RBM treated, CIT treated, and AIT treated titanium implants are 10.8 ± 3.7 Ncm, 17.5 ± 3.5 Ncm, and 28.1 ± 2.4 Ncm, respectively. The findings of the study indicate the cyclic precalcification in an effective surface treatment method that would help accelerate osseointegration and impart bioactive property of titanium implants.

## 1. Introduction

Titanium and titanium alloys are commonly used as prosthetic materials in dental or orthopedic surgeries because of their ability to offer a better corrosion resistance and excellent biocompatibility and to serve as a base for the deposition of hydroxyapatite (HAp) coating, which would further impart the bioactive properties when they are implanted in the human body. The high corrosion resistance and excellent biocompatibility of titanium/titanium alloys originate from the naturally formed thin TiO_2_ layer (thickness: ~4–6 nm). The TiO_2_ layer possesses a chemically and thermodynamically stable structure; it has low solubility, and it did not show any toxicity under* in vivo* conditions. However, one of the major limitations in using titanium and titanium alloys as implant materials is their poor osseointegration property. It has been reported that it would take several months to more than a year to achieve osseointegration due to their bioinert characteristics compared to those with better bioactivity [1]. For these reasons, the recent trend in dental implant research and development is to engineer the surface property of titanium/titanium alloy to achieve a better osseointegration [2–4]. The topography and morphology of the titanium implant surface can be changed with grit blasting, acid etching, anodization, and so on, and it has been reported in the literature that these surface treatments affect the adhesion and proliferation of osteoblasts to varying degrees [5–7]. With the advent of nanotechnology, generation of TiO_2_ nanotubes by anodization as one of the surface treatment methods has been evolved. The TiO_2_ nanotubes have been shown to accelerate the osseointegration of titanium implants. It has been reported that differentiation and proliferation of osteoblastic cells and mesenchymal stem cells and an enhanced osseointegration are facilitated on the nanostructured surface obtained by anodization [8–12].

Precalcification treatment that involves immersion of implants in calcium phosphate (the major elements of HAp) solutions has been explored as one of surface treatment methods to modify the surface of titanium implants so as to impart the bioinert characteristics and to improve their bioactivity. This method induces acid-base reactions between the TiO_2_ surface layer and the ions, and it has been reported to accelerate the biomimetic deposition of apatite in the simulated body fluid [13, 14].

In this study, cyclic precalcification of titanium implants is performed by immersing the implants in calcium phosphate solution after establishing the nanotubular TiO_2_ layer by anodization in order to improve the biocompatibility and the bioactivity properties of the implants. The impact of these surface treatments on the bioactivity of the implant was tested using simulated body fluid and the tibias of rats.

## 2. Materials and Methods

### 2.1. Preparation of Dental Implants

**Figure 1:**
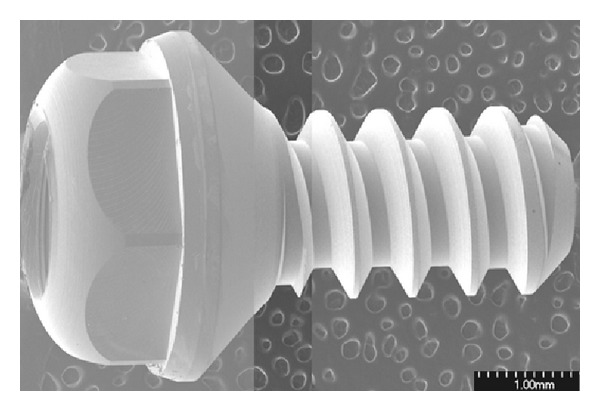
SEM image of an experimental titanium implant specimen.

In the present study, titanium implants have a dimension of 4 mm in length and 2 mm in diameter (KJMEDTECH Co., Ltd., Korea), which were fabricated from 5 mm *Ø*  titanium rods (Kobe Steel Ltd., Japan) (Figure 1). Eighteen implants were used in the study, and they were divided into three groups. The first group of implants was subjected to blasting using resorbable blasting media, and they were termed as RBM treated. The second group of implants was subjected to RBM treatment followed by anodizing and continuous immersion treatment which was termed as CIT. The third group of implants was subjected to RBM treatment followed by anodizing and alternate immersion treatment, and they were termed as AIT. The details of each type of treatment were addressed in the following sections. The classification of various groups used in this study is listed in Table 1.

### 2.2. RBM Treatment

Two types of bioabsorbable HAp powders (MCD powder, Hi-Med, USA) having an average diameter of 100–150 *μ*m and less than 90 *μ*m were mixed in 50/50 wt% and used for RBM treatment. The HAp powder mixture was blasted onto the implant surface at 4 barometric pressure. The HAp-blasted surface was subsequently acid-etched with 20% HNO_3_ for 10 min, washed with deionized water, and ultrasonically cleaned in acetone and alcohol solution for 5 min. All RBM treated implants were kept inside a desiccator for more than 24 h at 50°C.

### 2.3. TiO_2_ Nanotube Formation

The RBM treated implants were acid-etched using HNO_3_ : HF : H_2_O (12 : 7 : 81) mixture for 10 s, washed 5 times by shaking in deionized water and then dried. The Ti implants were used as the anode, while a large platinum plate served as the cathode, and they were connected by a DC regulator (Kwangduck FA, Korea). Anodic oxidation was performed using an electrolyte solution that contains glycerol, 20 wt% H_2_O and 1 wt% NH_4_F at 20 mA/cm^2^, pulsed at 20 V for 60 min. After anodization, the Ti implants were ultrasonically cleaned in deionized water for 1 min, and they were kept inside a desiccator for more than 24 h at 50°C. The microstructure of the anodized implant at the surface as well as at horizontal and vertical cross sections was examined by a field emission scanning electron microscopy (FE-SEM; S800, Hitachi, Japan).

### 2.4. Precalcification Treatment

Precalcification treatment was performed on RBM treated followed by anodized implants by immersing them in 0.05 M NaH_2_PO_4_ solution saturated with Ca(OH)_2_ to induce acceleration of HAp deposition. One group of implants was initially immersed in 0.05 M NaH_2_PO_4_ solution at 80°C for 20 min followed by immersion in saturated Ca(OH)_2_ solution at 100°C for 20 min (termed as CIT treated). The other group of implants underwent a cyclic immersion treatment for 20 cycles each consisting of the following four steps: (1) immersion in 0.05 M NaH_2_PO_4_ solution at 80°C for 1 min; (2) immersion in deionized water at 25°C for 5 s; (3) immersion in Ca(OH)_2_ saturated solution at 100°C for 1 min; and (4) immersion in deionized water at 25°C for 5 s (termed as AIT treated). After precalcification treatments, the CIT and AIT treated Ti implants were heat treated in an electric furnace (Ajeon Industrial Co., Ltd., Korea) for 2 h at heating rate of 10°C/min up to 500°C to stabilize the structure of the nanotubular TiO_2_ layer and to remove the impurities.

### 2.5. Apatite Formation Rate

To investigate the bioactivity of surface modified titanium implants, they were immersed in simulated body fluid (SBF), which has a pH and mineral composition similar to that of human plasma for 3 days. The SBF was prepared by adding 0.185 g/L of calcium chloride dihydrate, 0.09767 g/L of magnesium sulfate, and 0.350 g/L of sodium hydrogen carbonate to a Hanks solution (H2387, Sigma Chemical Co., USA), and the pH of the solution was regulated at 7.4 by adding small quantities of 1 N HCl. The surface modified titanium implants were treated in an autoclave at 120°C for 20 min and subsequently immersed in SBF, which was kept in an incubator regulated at 37°C with 5% CO_2_ for 3 days. The deposition of HAp was examined by FE-SEM, and the concentration of elements presented on the surface layer was analyzed by energy dispersive X-ray spectroscopy (EDS, Bruker, Germany).

### 2.6. Implantation Procedure

**Figure 2:**
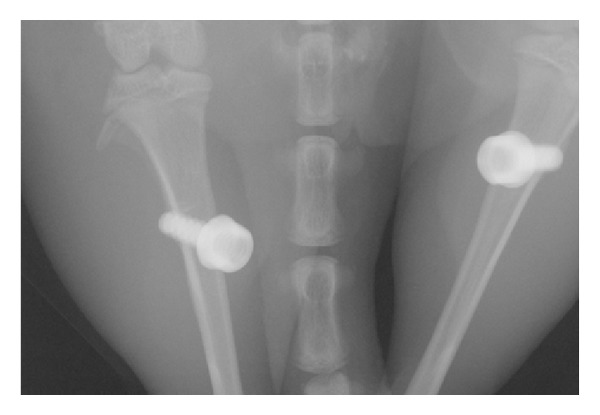
Micro-CT image of the rat tibias with the titanium implants.

Nine male Wistar rats (12-week-old and weighing 200 to 225 g) were used for animal test. A general anesthesia was performed using ketamine/xylazine (80 to 100 mg/kg and 10 to 20 mg/kg, resp.). Additionally, 2% lidocaine solution containing epinephrine (1 : 100,000) was also administered for local anesthesia. The surgical site was shaved and disinfected with a betadine scrub. This was followed by the elevation of full-thickness mucoperiosteal flaps. The implants were placed bilaterally in the distal tibial diaphysis using a self-tapping process. The micro-CT image of the rat tibias after implantation is shown in Figure 2. Resorbable sutures were used to approximate the surgical wound. Postoperatively, amoxicillin (6 mg/kg), an antibiotic, and nabumetone (5 mg/kg), a nonsteroidal anti-inflammatory drug, were administered orally by dissolving them in the drinking water. Postoperatively, amoxicillin (1 mg/kg) an antibiotic and ketoprofen (5 mg/kg), an analgesic, were administered intramuscularly. Four weeks later, the rats were sacrificed with an overdose of thiopental.

### 2.7. Removal Torque Measurements

The removal torque values were measured after 4 weeks of postimplantation. The implantation sites in the rat tibia were surgically exposed via a sharp dissection to bone and clinically examined after carefully removing the overgrown bone and soft tissues. Removal torque tests were performed for all implants (six implants per group) in the tibia using a digital torque gauge (9810P, Aikoh Engineering Co., Japan). The Tukey test was used to compute *P* values to estimate the differences in removal torque values among the three groups. *P* < 0.05 was considered as statistically significant. The surfaces of removed implants were examined to evaluate the level of osseointegration by FE-SEM, and the concentration of elements present on the surface was analyzed by EDS.

## 3. Results

### 3.1. Structure of the Nanotubular TiO_2_ Layer on Dental Implants

**Figure 3:**
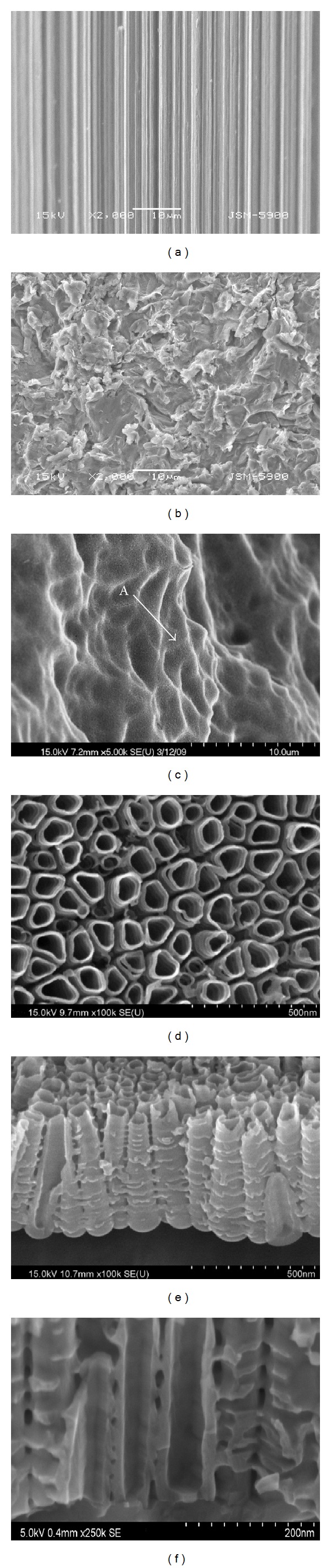
SEM images of implant surfaces of screw-type implants. (a) Machine turned (×2 K), (b) RBM treated (×2 K), (c) anodized after RBM treatment (×5 K), (d) magnification of point A (×100 K), (e) laterally fractured after anodic oxidation treatment (×100 K), and (f) nanotubes cut laterally.

Figure 3 shows the scanning electron micrographs of the machine-turned, the RBM treated, and the anodized surfaces of the titanium implants. The scoring marks generated during machining, which are parallel to the machining direction, are evident on the machined samples (Figure 3(a)), whereas a rough and irregular pattern is observed on the RBM treated surface (Figure 3(b)). The surface microstructure of the TiO_2_ layer obtained by anodizing is characterized by an irregular pattern (Figure 3(c)). However, a closer examination of the surface at high magnification (at the point marked as “A” in Figure 3(c)) clearly reveals the formation of self-organized nanotubes with 125.4 ± 11.2 nm and 83.5 ± 6.5 nm in diameter (Figure 3(d)). In order to have a better understanding of the architecture of the nanotubes, they are fractured horizontally and cut vertically. The fractured and cut surface clearly reveals that each nanotube possesses a different structure and the diameter of each of them is increased towards the bottom of the tubes. The nanotubes are rather empty in the inner side, and they have an average length of 592.6 ± 29.3 nm.

### 3.2. Formation of Apatite on the CIT and AIT Treated Nanotubular TiO_2_ Layer

**Figure 4:**
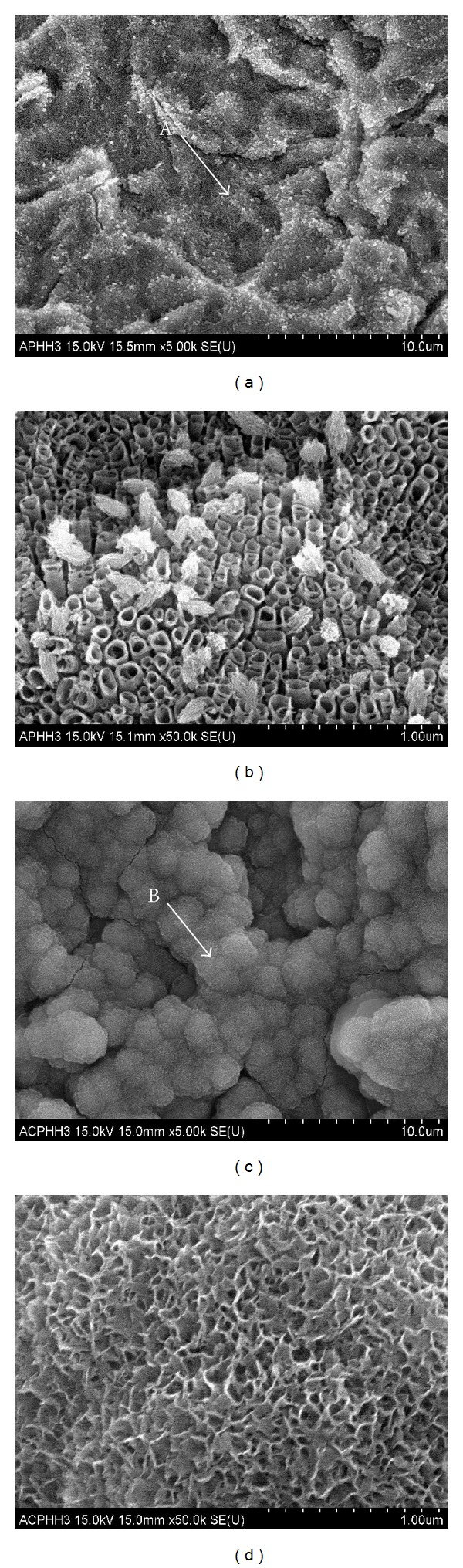
FE-SEM images after immersion in the SBF solution for 3 days. (a) Noncyclic-precalcified (CIT) group; (b) magnification of point A; (c) cyclic-precalcified (AIT) group; (d) magnification of point B.

Figure 4 shows the scanning electron micrographs of CIT and AIT treated titanium implants after immersion in SBF for 3 days. The nanotubular structures with some deposits are observed in the CIT groups but spur-shaped structures are evident on surfaces of AIT treated titanium implants, suggesting the early stage of apatite deposition.  Table 2 shows the results of the EDS analysis which indicate that the amount of Ca and P on the surface of AIT treated titanium implants are significantly higher than those formed on CIT treated titanium implants.

### 3.3. Analysis of the Removal Torque and Surfaces of the Removed Implants

**Figure 5:**
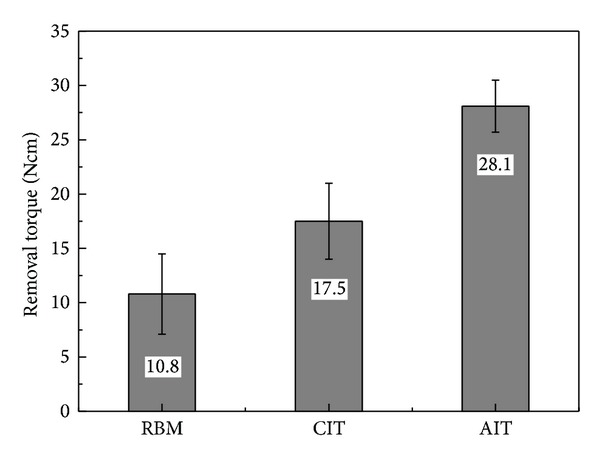
Removal torque value (Ncm) after implantation for 4 weeks.

Figure 5 shows the results of removal torque analysis performed on RBM treated, CIT treated, and AIT treated titanium implants when they are removed from the rat tibias after 4 weeks of implantation. The removal torque values of the RBM treated, CIT treated and AIT treated titanium implants are 10.8 ± 3.7 Ncm, 17.5 ± 3.5 Ncm, and 28.1 ± 2.4 Ncm, respectively, and there is a statistically significant difference in the removal torque value between these groups (*P* < 0.01).

**Figure 6:**
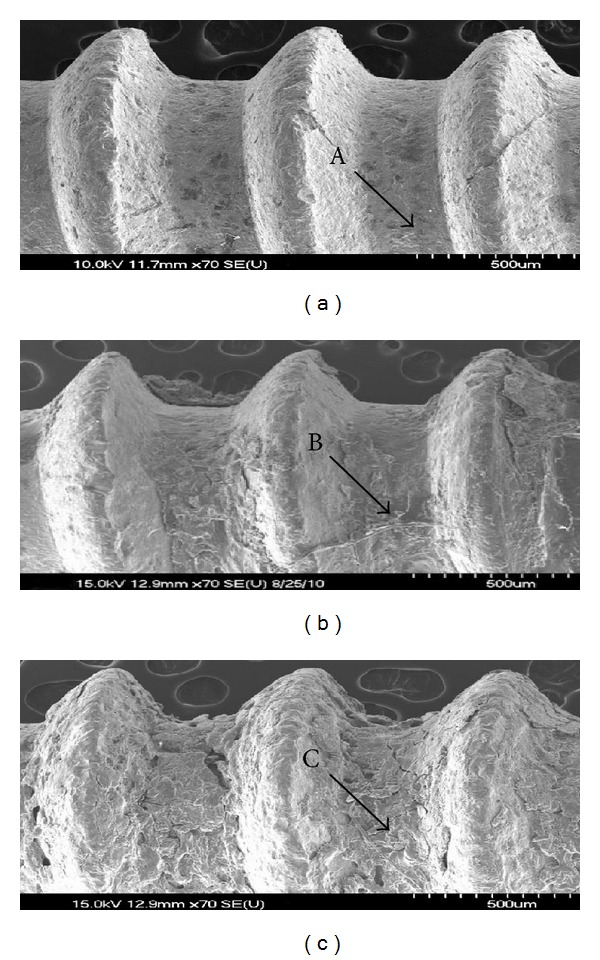
FE-SEM images of the surfaces of removed implants. (a) RBM treated; (b) noncyclic-precalcified (CIT) group; (c) cyclic-precalcified (AIT) group. (A, B, and C are the positions for EDS in Table 3).

Figure 6 shows the scanning electron micrographs of the implant surface after performing the removal torque measurements. The surface of the RBM treated titanium implants exhibits fractures at the interface between the newly formed bones and the implant. However, only the fracture of the newly formed bones could be observed on AIT treated titanium implants. On the other hand, the CIT treated titanium implants reveal fractures on the newly formed bones as well as at interface between the between the newly formed bones and the implant.

Table 3 shows the EDS analysis results performed at the surface of the explanted implants after 4 weeks of implantation in the rat tibias. The amounts of Ca and P (measured at the point marked as “C” in Figure 6(c)) are relatively higher for the AIT treated titanium implants which showed agglutination and fractures in the newly formed bones. In contrast, the RBM treated (measured at the point marked as “A” in Figure 6(a)) and CIT treated titanium implants (measured at the point marked as “B” in Figure 6(b)) both showed fractures at the interface, exhibit a relatively lower amounts of Ca and P.

## 4. Discussion

The methods for formation of micro/nanostructure on the implant surface assume significance as they aid in osseointegration. It has been reported that a nanostructured surface favors osseointegration as it provides a wider surface area than the microstructured surface [15]. It has been reported that the mechanism of generation of nanotubular TiO_2_ on the surface of the titanium is due to a dynamic equilibrium between the electrochemically assisted growth of the oxide layer and the simultaneous etching/dissolution of the oxide layer by the fluoride ions present in the electrolyte [16, 17]. The anodization methodology offers an extended window of opportunity in terms of its ability to control the growth of the oxide layer by an appropriate choice of a variety of aqueous and organic electrolytes, presence of additives, pH of the electrolyte, applied voltage and/or current, and treatment time and to fabricate the TiO_2_ nanotubes. In addition, it is a simple and cost-effective surface engineering approach when compared to other surface treatments used for titanium implants [17, 18]. The anodized titanium implants prepared in the present study exhibit the formation of self-organized TiO_2_ nanotubes consisting of nanotubular structure with two different diameters. The mean diameter of the large nanotubes is 125.4 ± 11.2 nm, whereas the diameter of the relatively smaller ones is 83.5 ± 6.5 nm. The mean length of the nanotubes is 592.6 ± 29.3 nm (Figure 3). The structural features assessed at the top surface and at the horizontal and the vertical cross sections reveal that each TiO_2_ nanotube is constructed in such a way that they are independent and stacked together with appropriate spacing between them. The diameter of the nanotubes is increased towards the bottom of the tube, and the interior of the tube is empty. This is due to the simultaneous and continuous etching of the oxide layer by the fluoride ions present in the electrolyte. The formation of such a structural feature that consists of a nanotube with an empty inner space can be used for loading various chemicals, ranging from small molecules to proteins, drugs, biomolecules, and so on. In addition, implantation of drug loaded nanotubes at specific targeted site would reduce the systemic side effects caused by the toxicity of drugs [19, 20]. In this study, the nanotubular TiO_2_ structures are generated on the titanium implants. In addition, they are subjected to precalcification treatment, which facilitates the deposition of Hap, imparts the bioactive property, and improves the activation level of the titanium implant* in vivo. *Precalcification is known to impart bioactivity to the surface of titanium implants, which already possess the bioinert characteristics and facilitates osseointegration [3, 21–23]. Feng et al. [23] have confirmed that deposition of HAp is facilitated by immersion in supersaturated calcium phosphate solution (SCP) after immersion and boiling in saturated Ca(OH)_2_ solution for 30 minutes when compared to the nontreated titanium implant. Ma et al. [24] have reported that immersion in the SCP solution for 2 weeks following precalcification, immersion in 0.5 M NaH_2_PO_4_ solution for 24 h, and immersion in saturated Ca(OH)_2_ solution for 5 h after creating the nanotubular TiO_2_ layer by anodization have resulted in spur-shaped HAp deposition, which is an early stage of HAp deposition. In addition, Kodama et al. [14] have reported that the deposition of HAp is rapidly accelerated after immersing in SBF for 10 days at 37°C following cyclic immersion in 0.02 M NaH_2_PO_4_ solution and saturated Ca(OH)_2_ solution at room temperature. In the present study, the CIT and AIT treated titanium implants are immersed in SBF for 3 days in order to study the effect of immersion conditions on the deposition of HAp coating. CIT treated titanium implants fail to show a clear deposition of HAp. In contrast, AIT treated titanium implants exhibit spur-shaped structural feature, which indicates an early stage of HAp deposition. In addition, the amount of Ca and P is relatively higher in AIT treated titanium implants, which indicates a better surface activity of these implants (Figure 4, Table 2).

Removal torque measurement is a biodynamic method to measure the resistance against rotational movements, which is often used to evaluate the healing process after the implantation of spiral implants. The results obtained with the use of this method can be influenced by the shape and surface structure of the implants, but it is widely accepted as an objective method to evaluate the recovery status of the bone-implant interface [25]. Carlsson et al. [26] have reported that the removal torque values measured after 6 weeks of implantation are relatively higher for implants having a rough surface than those having a smooth surface. In the present study, the titanium implants are placed in the rat tibias, and the removal torque values of the RBM treated, CIT treated and AIT treated titanium implants are 10.8 ± 3.7 Ncm, 17.5 ± 3.5 Ncm, and 28.1 ± 2.4 Ncm, respectively, and there is a statistically significant difference in the removal torque value between these groups (*P* < 0.01; Figure 5). Examination of the surface features of the implants after explantation reveals fractures at the interface between the newly formed bones and the implant surface due to weak adhesion in the machine turned and RBM treated implants. In contrast, agglutination and fractures inside the newly formed bones are observed for CIT treated titanium implants. This is because of the strong adherence of the implant surface with the newly formed bone. In addition, for CIT treated titanium implants, the surface of the implants after explantation reveals higher amounts of Ca and P (Figure 6, Table 3). The inferences of the present study confirm that it is possible to change the microstructural features of the surface of RBM treated titanium implants by generating a nanotubular structure by electrochemical anodization using a glycerol based electrolyte containing appropriate concentrations of fluoride ions. In addition, the findings of the study reveal that it would be possible to impart bioactivity to the surface of titanium implants that has already possessed the bioinert characteristics by cyclic precalcification treatment using NaH_2_PO_4_ and saturated Ca(OH)_2_ solutions.

## 5. Conclusion

This study is performed with an objective to modify the surface of titanium implants to increase its bioinert and biocompatibility properties by anodizing and to impart bioactive characteristics to increase their activation level *in vivo *by precalcification treatment. Anodizing of the titanium implants enables the formation of self-organized TiO_2_ nanotubes consisting of nanotubular structure with two different diameters. Cyclic precalcification treatment imparts a better bioactive property and enables an increase in activation level of the titanium implants. The removal torque value of the AIT treated titanium implants is significantly higher than any other group (*P* < 0.01). The study concludes that cyclic precalcification in an effective surface treatment method to impart bioactive property and implementation of such a methodology on nanotubular TiO_2_ coated titanium implants obtained by anodization will be a useful approach in developing implant materials of the future.

## Figures and Tables

**Table 1:** Classification of groups used in this study.

Group	Method
Untreated	As machined
RBM-treated	Sand-blasted by RBM
CIT	RBM + anodizing + continuous immersion treated
AIT	RBM + anodizing + alternating immersion treated repeatedly 20 times

**Table 2:** EDS results after immersion in SBF for 3 days (wt%).

Group	Ti	O	C	Ca	P	Mg
CIT	50.6	45.4	1.8	1.5	0.3	—
AIT	9.6	46.2	4.0	25.1	14.4	0.7

**Table 3:** EDS results of the surfaces of the removed implants, which were placed in the rat tibia for 4 weeks (wt%).

Group	Ti	O	C	Ca	P	Mg
RBM treated (point A in Figure 6)	61.8	25.3	6.7	3.8	2.0	—
CIT (point B in Figure 6)	18.5	42.7	19.7	12.0	6.9	0.2
AIT (point C in Figure 6)	—	41.6	15.4	29.0	13.6	0.4
